# 

**Published:** 1889-04

**Authors:** 


RECEIPTED FOR 1888—J E Rogers, M V B Miler, J B Rutland, Jas L Smyth, E R Rollins, T L Christian, M E Tilton, F H Miller, Robt L Jones, Edward Pitts.

FOR 1889—N B Warren, A T Park, J H Henry, D Lindley, C C Hart to June, J V Plummer, H Perdue, E H Parham.

FOR 1890—J W Saunders, to July, Robt E Morgan, F H Willerby.

